# Association between serum sodium trajectory and mortality in patients with acute kidney injury: a retrospective cohort study

**DOI:** 10.1186/s12882-024-03586-y

**Published:** 2024-05-02

**Authors:** Shanhe Huang, Xiaojing Li, Baorong Chen, Yaqi Zhong, Yuewei Li, Tucheng Huang

**Affiliations:** 1 Zhongshan School of Medicine, Sun Yat-Sen University, Guangzhou, Guangdong China; 2 Department of Emergency, the Eighth Affiliated Hospital of Sun Yat-Sen University, Shenzhen, China; 3 Huangpu Customs International Travel Health Care Center, Shenzhen, Guangdong China; 4 Department of Pulmonary and Critical Care Medicine, Sun Yat-Sen Memorial Hospital, Sun Yat-Sen University, Guangzhou, Guangdong China; 5 Department of Cardiology, Sun Yat-Sen Memorial Hospital, Sun Yat-Sen University, 107 Yanjiang West Road, Guangzhou, Guangdong 51000 China; 6 Laboratory of Cardiac Electrophysiology and Arrhythmia in Guangdong Province, Guangzhou, China

**Keywords:** Acute kidney injury, Mortality, Serum sodium trajectory, Cohort study, MIMIC database

## Abstract

**Introduction:**

Dysnatremia is strongly associated with poor prognosis in acute kidney injury (AKI); however, the impact of sodium trajectories on the prognosis of patients with AKI has not yet been well elucidated. This study aimed to assess the association between sodium trajectories in patients with AKI and mortality at 30-day and 1-year follow-up.

**Methods:**

This retrospective cohort study used data from Medical Information Mart for Intensive Care (MIMIC)-IV database, and patients diagnosed with AKI within 48 h after admission were enrolled. Group-based trajectory models (GBTM) were applied to map the developmental course of the serum sodium fluctuations. Kaplan–Meier survival curve was used to compare differences in mortality in AKI patients with distinct serum sodium trajectories. Hazard ratios (HRs) were calculated to determine the association between trajectories and prognosis using Cox proportional hazard models.

**Results:**

A total of 9,314 AKI patients were enrolled. Three distinct sodium trajectories were identified including: (i) stable group (ST, in which the serum sodium levels remained relatively stable, *n* = 4,935; 53.0%), (ii) descending group (DS, in which the serum sodium levels declined, *n* = 2,994; 32.15%) and (iii) ascending group (AS, in which the serum sodium levels were elevated, *n* = 1,383; 14.85%). There was no significant difference in age and gender distribution among the groups. The 30-day mortality rates were 7.9% in ST, 9.5% in DS and 16.6% in AS (*p* < 0.001). The results of 1-year mortality rates were similar (*p* < 0.001). In adjusted analysis, patients in the DS (HR = 1.22, 95% confidence interval [CI], 1.04–1.43, *p* = 0.015) and AS (HR = 1.68, 95% CI, 1.42–2.01, *p* = 0.013) groups had higher risks of 30-day mortality compared to those in the ST group.

**Conclusion:**

In patients with AKI, the serum sodium trajectories were independently associated with 30-day and 1-year mortality. Association between serum sodium level trajectories and prognosis in patients with AKI deserve further study.

## Introduction

Acute kidney injury (AKI) is frequently encountered in the intensive care unit (ICU) setting, marked by an abrupt deterioration in renal function and diminished urine output, which can disrupt the electrolytes and acid–base balance, cause fluid overload, and impact the function of other organ systems [1]. AKI occurs in approximately 30–50% of ICU patients [2] and is independently associated with in-hospital and long-term adverse outcomes [3], resulting in significant socioeconomic burden.

Electrolyte disturbance, among which serum sodium (sNa) disorders like hyponatremia (sNa < 135 mEq/L) and hypernatremia (sNa > 145 mEq/L) are awfully common [4, 5], is often observed in patients with AKI. Dysnatremia, a term used to describe both hyponatremia and hypernatremia, occurs in approximately 25% of ICU patients and has been shown to be associated with elevated mortality of hospitalized patients [6–8]. However, these studies mainly focused on the effect of sNa on admission or discharge on patient prognosis. Owing to the fact that the conditions of ICU patients may change rapidly, the trajectory of sNa fluctuation in patients after admission, rather than the absolute value of sNa at a specific time point, may be more clinically valuable for monitoring prognosis. The predictive effect of sNa trajectories on patient prognosis has been reported in some studies [9–12]. For instance, Jonathan et al. reported that distinct sNa trajectories were significantly associated with higher in-hospital mortality and kidney replacement therapy. However, most studies on sNa trajectories set their cut-off point between different groups subjectively, lacking the necessary objectivity and robustness required in such studies. Group-based trajectory modeling (GBTM), a novel statistical strategy first introduced by Chewcharat et al. [13], could provide more objective and robust classification criteria between different groups, which had been regarded as a widely accepted standard recently.

Collectively, we hypothesized that different in-hospital sNa trajectories identified by GBTM were associated with different 30-day and 1-year mortality outcomes. Thus, in this study, we grouped AKI patients according to their sNa trajectories using GBTM and explored the impact of different sNa trajectories on patient mortality at 30-day and 1-year follow-up. We aimed to elucidate the effect of sNa fluctuation patterns on the prognosis of AKI patients.

## Materials and methods

### Data source

The data analyzed in this study were obtained from the publicly accessible Medical Information Mart for Intensive Care IV (MIMIC-IV) database, which contains de-identified health information of patients admitted to the ICU of the Beth Israel Deaconess Medical Center in the USA from 2008 to 2019. The database comprises comprehensive data, including demographic information, clinical features, laboratory results, medication details, and vital signs. Data for analysis were extracted from the institutional electronic health record system, using the Structured Query Language (SQL). The author Yuewei Li has completed the Collaborative Institutional Training Program exam (certification number: 10007248) and the study has received institutional review board (IRB) approval. Informed consent is not necessary for the secondary utilization of this de-identified database.

### Study population

Patients diagnosed with AKI within 48 h after ICU admission as per the Kidney Disease Improving Global Outcomes (KDIGO) criteria [14] were enrolled. The exclusion criteria were as follows: (i) patients with missing data of sNa for the seven consecutive days after ICU admission; (ii) patients with age younger than 18 years; (iii) patients with less than 48 h of ICU stay; (iv) patients receiving maintenance dialysis treatment. For patients hospitalized for more than 7 days, only the first 7 days of hospitalization were analyzed for sNa measurements. In patients admitted to the ICU multiple times, only the first admission was included in the analysis.

Base on the Group-Based Trajectory Modeling (GBTM), we divided the participants into three groups. In a study exploring the link between sodium level changes over a week and subsequent survival periods, the use of Group-Based Trajectory Modeling (GBTM) begins with comprehensive data collection and organization. This initial stage covers not only the tracking of sodium levels daily for a week but also observing survival times during follow-up. At this juncture, the accuracy, error checking, and management of missing or abnormal data are imperative to ensure the reliability of future analyses.

Subsequently, GBTM analyzes patient data, primarily to categorize patients based on the dynamic shifts in their sodium levels. This technique discerns typical patterns of sodium variation, essentially having patients grouped by their sodium level changes. In the implementation of GBTM, specific polynomial shapes are predetermined, exploring models ranging from two to six groups. The selection of the optimal number of groups involves an iterative process, evaluating model fitting criteria such as the Akaike Information Criterion (AIC) and the Bayesian Information Criterion (BIC), along with assessments of group similarities and differences.

This detailed analytical method successfully outlines various sodium level trajectories, clearly representing the diverse trends within the study population over time. Each trajectory group embodies a distinct sodium fluctuation pattern, including but not limited to stability, and gradual increases or decreases. Through delineating these unique trajectory groups, researchers can further examine the association between specific patterns of sodium changes and patient survival times, thereby offering novel insights into the effects of sodium levels on patient prognosis. The methodology outlined utilizes the R package “traj.”

### Data collection

Baseline data mainly included demographic information (age, gender), vital signs [systolic blood pressure (SBP), diastolic blood pressure (DBP), mean blood pressure (MBP), respiratory rate (RR), heart rate (HR) and oxygen saturation (SpO_2_)], glucose, white blood cell count (WBC), hemoglobin (HB), platelet, sodium, potassium, chloride, serum creatinine (SCr), anion gap (AG), blood urea nitrogen (BUN), international normalized ratio (INR), prothrombin time (PT), partial thrombin time (PTT), albumin, multimorbidity (sepsis, shock, heart failure, respiratory failure, hypertension, diabetes mellitus, chronic kidney disease (CKD) and pneumonia etc.), Risk assessment scores [simplified acute physiology score II (SAPS II), sequential organ failure assessment (SOFA) and Oxford acute severity of illness (OASIS)]. Baseline SCr was defined as the minimum value of SCr within 7 days prior to admission or, if the baseline value was unavailable, as the first SCr measurement at admission [15], The main predictor was the in-hospital sNa trajectory, which was assessed for each patient based on the sNa values during the hospital stay.

### Definition and outcomes

AKI was diagnosed according to KDIGO criteria [14]: (1) SCr increased by 0.3 mg/dL (or ≥ 26.5 μmol/L) within 48 h; or (2) increased by ≥ 1.5-fold from baseline within the prior 7 days; and/or (3) a decrease in urine output (UO) < 0.5 ml/kg/h for 6 h. The primary outcome was 30-day mortality and secondary outcomes was 1-year mortality.

### Statistical analysis

For continuous variables, the Shapiro–Wilk test was used to determine whether the data were normally distributed, thus those normally distributed were presented as mean ± standard deviation, otherwise median (with interquartile range (IQR)). Categorical variables were presented as absolute counts (percentages). Comparisons between groups were performed using the Student’s *t* test for continuous variables, and chi-square test or Fisher’s exact test for categorical variables. A two-tailed *p* value of less than 0.05 was considered statistically significant.

**Fig. 1:**
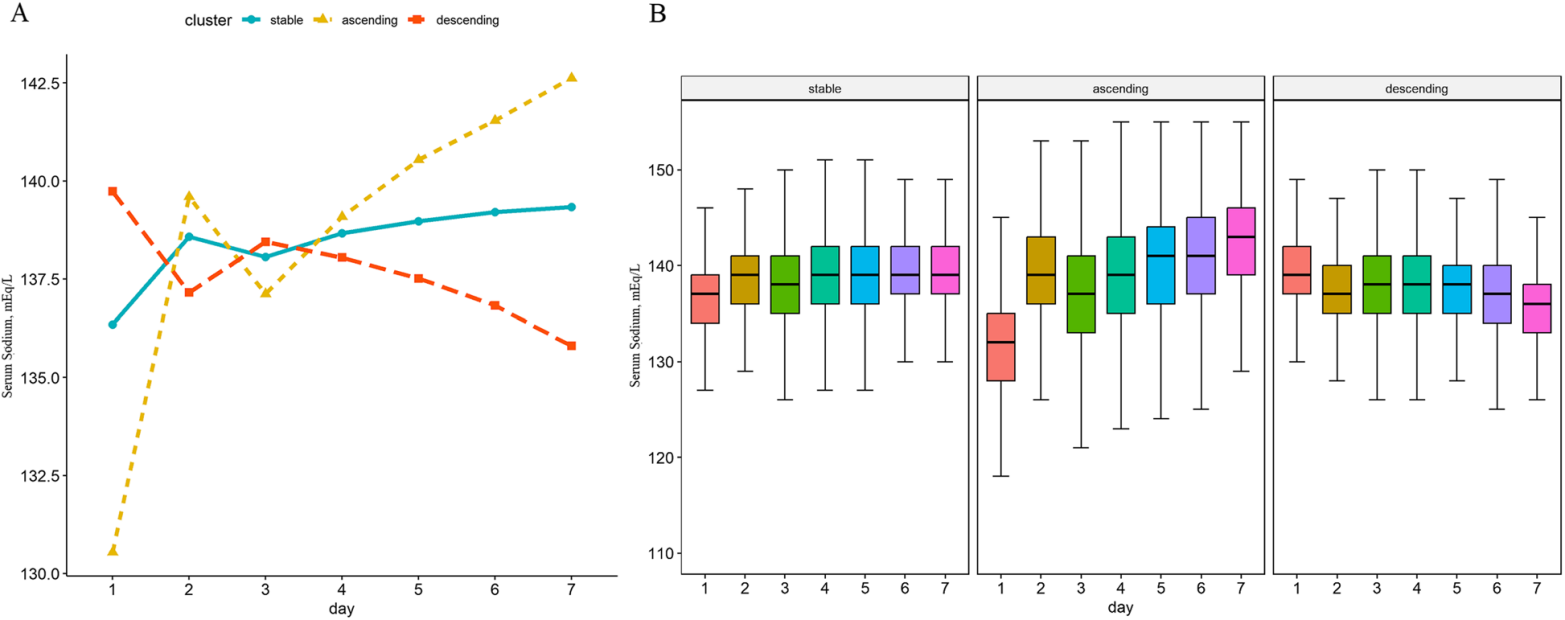
Identification of serum sodium trajectories. **A** The average Serum sodium level trajectories of patients in different group with acute kidney injury (AKI). **B** The mean serum sodium levels0 from day 1 to day 7 in each group

SNa trajectories during the first seven days of ICU admission were created by GBTM to group longitudinal measurements intro inter-related subgroups. GBTM considers the patterns of change for measures across multiple time points and identifies distinctive trajectories, allowing for a more robust and objective classification compared to subjective criteria. The complete algorithm was fully described elsewhere. Briefly, GBTM predicts the trajectory for each group, estimates the probability of each individual of group membership, and assigns them to the group based on their highest probabilities which were summarized by a finite set of different polynomial functions of time. The model with the highest number of fitting categories was selected based on the Bayesian information criterion (BIC). Aiming to ensure that each group had a clear clinical interpretation and utility, we referred to the clinical experience of experts in the field and sought to find a proper model reflecting clinically meaningful distinctions among patient trajectories in each group. We also assessed the stability and reliability of our chosen model across different samples to ensure that the model groups were not only statistically optimal but robust and reliable in clinical practice. Hence, after thoroughly considering BIC scores, clinical experience, and the additional criteria above, sNa trajectories were categorized into three main trajectories: (i) stable group (ST), where sNa levels remained relatively stable, (ii) descending group (DS), where sNa levels declined, and (iii) ascending group (AS), where sNa levels were elevated. All trajectories are depicted in Fig. 1.

Kaplan–Meier survival analysis with the log-rank test were conducted to compare the 30-day mortality and 1-year mortality among distinct sNa trajectories. Furthermore, Cox proportional hazard models were performed to calculate the hazard ratios (HRs) and 95% confidence intervals (CIs) assess the association between sNa trajectories and 30-day mortality of AKI patients. Model 1 was adjusted for age, gender, BMI, BP, HR and SpO_2_, and model 2 was further adjusted for CKD, hypertension, diabetes, heart failure, sepsis, stroke, AKI stage, SAPSII and OASIS scores. Model 3 extended model 2 by further adjusting mean sNa and diuretics use. In the subgroup analyses, AKI patients were stratified by age, gender, hypertension, diabetes, heart failure, CKD, sepsis, vasopressors and diuretics.

GraphPad Prism 8.0 (GraphPad Software, Inc, La Jolla, CA, USA) and RStudio (version 1.0.143) were used for data analysis in the study.

## Results

### Baseline characteristics

**Fig. 2:**
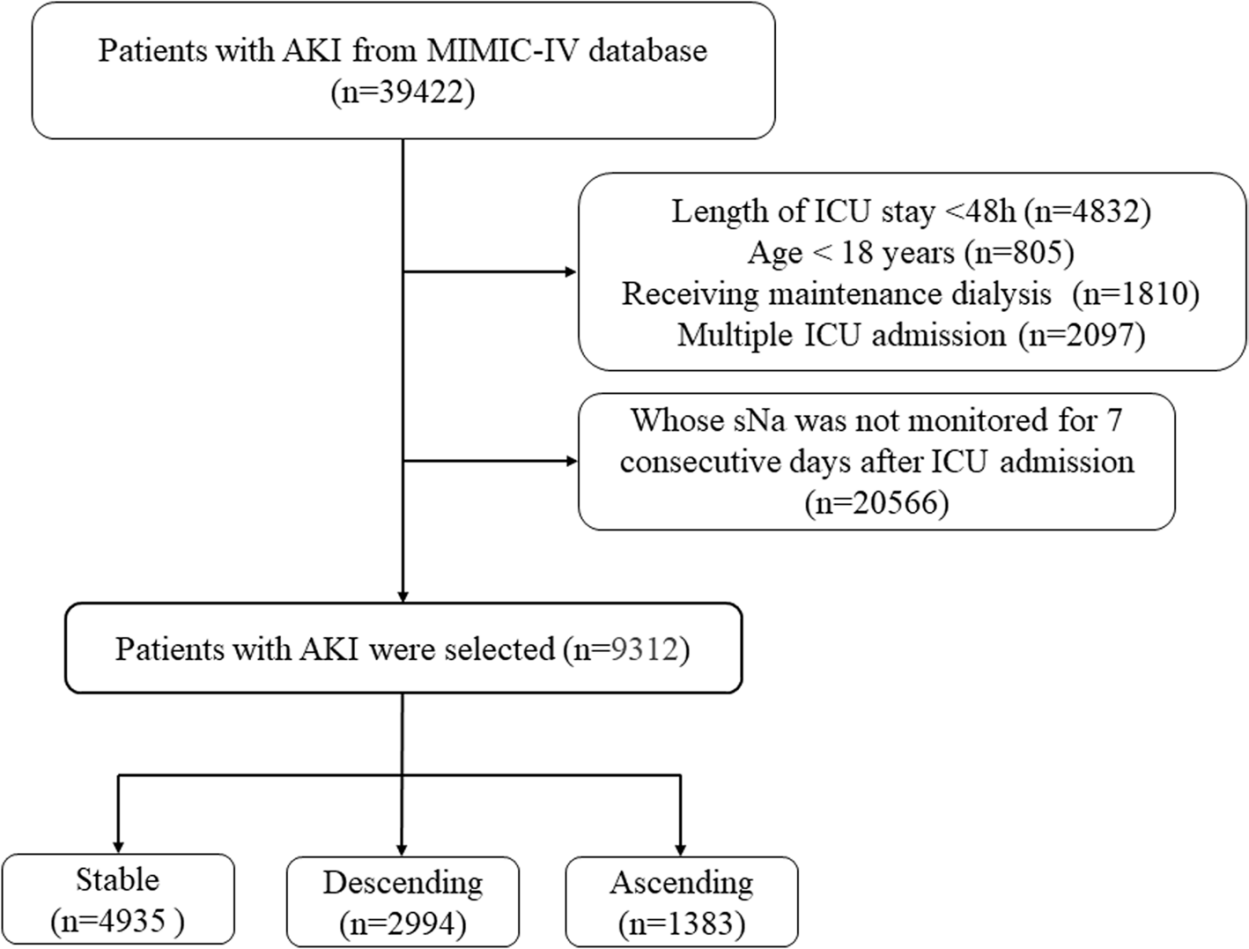
Flowchart depicting the inclusion of study population

In the present study, a total of 9,314 AKI patients were enrolled from the MIMIC-IV database. The flowchart shown in Fig. 2 outlines how patients were selected for the study. Patients were classified into the most probable trajectory pattern in the light of high probability by GBTM. There were 4,935 (53.0%), 2,994 (32.15%) and 1,383 (14.85%) participants in ST, DS and AS groups, respectively. The groups were well balanced in terms of age and gender distribution. In terms of comorbidities, the DS group had the highest prevalence of ARDS, while the AS group had the highest prevalence of sepsis and cirrhosis. The ST group had the lowest prevalence of all comorbidities. In addition, the patients of each group also differed significantly in their admission sNa, lowest sNa, and highest sNa, as well as their mean sNa during the hospital stay. The DS group had the lowest admission, lowest, and mean sNa levels, while the AS group had the highest admission, highest, and mean sNa levels. The ST group had intermediate sNa levels. Although more patients in AS group received diuretics (62.6%, 59.1% versus 65.4% in ST, DS versus AS group, respectively, *p* < 0.001), there was no significant difference in furosemide dosage between groups (Table 1).

**Table 1:** Baseline characteristics of patients stratified by sodium trajectory groups

	All (*n* = 9,312)	Stable (*n* = 4,935)	Descending (*n* = 2,994)	Ascending (*n* = 1,383)	*p*
Male	5,250 (56.4%)	2,784 (56.4%)	1,676 (56%)	790 (57.1%)	0.776
Age, yr	66.8 (55.5, 76.6)	67.1 (55.7, 76.7)	66.7 (55.4, 76.7)	65.8 (55.0, 76.5)	0.370
BMI, kg/m^2^	27.9 (24.0, 32.8)	28.0 (24.1, 33.0)	27.6 (23.9, 32.5)	28.0 (24.1, 33.1)	0.042
HR, bpm	86.1 (75.4, 98.5)	86.3 (75.9, 98.5)	85.2 (74.7, 97.1)	87.5 (75.7, 100.7)	< 0.001
SBP, mmHg	114.6 (105.2, 127.3)	113.9 (105.0, 126.0)	116.4 (106.4, 129.6)	112.8 (103.7, 126.5)	< 0.001
DBP, mmHg	61.7 (55.3, 69.3)	61.5 (55.3, 69.1)	62.2 (55.7, 70.2)	61.0 (54.6, 68.1)	< 0.001
MBP, mmHg	76.6 (70.6, 84.7)	76.3 (70.5, 84.4)	77.7 (71.2, 86.2)	75.5 (69.6, 82.5)	< 0.001
RR, bpm	19.1 (16.8, 22.0)	19.0 (16.8, 22.0)	18.8 (16.6, 21.7)	19.8 (17.3, 23.1)	< 0.001
SpO_2_, %	97.1 (95.7, 98.5)	97.1 (95.7, 98.5)	97.2 (95.7, 98.6)	97.1 (95.6, 98.5)	0.276
**Comorbidities, n (%)**					
CKD	2023 (21.7%)	1083 (21.9%)	639 (21.3%)	301 (21.8%)	0.819
Hypertension	6030 (64.8%)	3189 (64.6%)	1944 (64.9%)	897 (64.9%)	0.958
Diabetes	2960 (31.8%)	1525 (30.9%)	965 (32.2%)	470 (34%)	0.077
Heart failure	2914 (31.3%)	1585 (32.1%)	920 (30.7%)	409 (29.6%)	0.142
ARDS	78 (0.8%)	331 (6.7%)	171 (5.7%)	112 (8.1%)	0.011
Sepsis	6333 (68%)	3269 (66.2%)	1963 (65.6%)	1101 (79.6%)	< 0.001
Stroke	1542 (16.6%)	713 (14.4%)	558 (18.6%)	271 (19.6%)	< 0.001
Ureteral obstruction	142 (1.5%)	76 (1.5%)	42 (1.4%)	24 (1.7%)	0.700
Cirrhosis	903 (9.7%)	425 (8.6%)	245 (8.2%)	233 (16.8%)	< 0.001
**Lab results**					
Mean sNa, mEq/L	138.5 (135.5, 141.0)	138.0 (135.5, 140.0)	140.0 (138.0, 143.0)	134.5 (130.0, 137.5)	< 0.001
Admission sNa, mEq/L	137.0 (134.0, 140.0)	137.0 (134.0, 139.0)	139.0 (137.0, 142.0)	132.0 (127.0, 135.0)	< 0.001
Lowest sNa, mEq/L	134.0 (131.0, 137.0)	135.0 (133.0, 137.0)	134.0 (131.0, 137.0)	131.0 (127.0, 135.0)	< 0.001
Highest sNa, mEq/L	142.0 (139.0, 145.0)	142.0 (139.0, 144.0)	141.0 (139.0, 144.0)	145.0 (141.0, 148.0)	< 0.001
Chloride, mEq/L	104.0 (100.0, 107.5)	104.0 (100.0, 107.0)	106.0 (102.5, 109.5)	100.0 (94.5, 104.5)	< 0.001
Potassium, mEq/L	4.2 (3.8, 4.6)	4.2 (3.8, 4.6)	4.1 (3.8, 4.5)	4.2 (3.8, 4.8)	< 0.001
AG	14.5 (12.5, 17.0)	14.5 (12.5, 17.0)	14.5 (12.5, 17.0)	15.5 (13.0, 18.0)	< 0.001
BUN, mg/dl	20.5 (14.0, 34.5)	20.0 (13.5, 32.5)	20.5 (14.0, 33.0)	24.0 (15.0, 43.0)	< 0.001
Blood glucose, mg/dl	133.2 (113.8, 161.9)	132.3 (113.0, 160.0)	132.2 (114.2, 159.3)	141.8 (116.7, 180.2)	< 0.001
Bicarbonate, mEq/L	22.5 (20.0, 25.0)	22.5 (20.0, 25.0)	23.0 (20.5, 25.5)	21.5 (18.5, 24.5)	< 0.001
Hemoglobin, g/dl	10.5 (9.1, 12.2)	10.5 (9.1, 12.2)	10.6 (9.2, 12.3)	10.4 (8.9, 12.2)	< 0.001
Hematocrit, %	31.9 (27.7, 36.8)	31.8 (27.6, 36.6)	32.2 (28.1, 37.2)	31.2 (26.9, 36.4)	< 0.001
Creatinine, mg/dl	1.0 (0.8, 1.6)	1.0 (0.8, 1.6)	1.0 (0.8, 1.6)	1.2 (0.8, 2.0)	< 0.001
PT, s	14.2 (12.6, 16.9)	14.2 (12.6, 16.8)	14.1 (12.6, 16.6)	14.6 (12.6, 18.4)	< 0.001
PTT, s	31.8 (27.5, 41.6)	31.8 (27.6, 41.6)	31.2 (27.3, 40.7)	32.4 (27.9, 43.5)	0.002
INR, s	1.3 (1.1, 1.6)	1.3 (1.1, 1.6)	1.3 (1.1, 1.5)	1.3 (1.1, 1.7)	< 0.001
WBC, K/uL	11.6 (8.4, 15.6)	11.6 (8.5, 15.6)	11.3 (8.3, 15.2)	12.1 (8.6, 17.1)	< 0.001
Platelets, K/uL	188.0 (132.0, 254.5)	189.5 (134.5, 257.0)	186.2 (132.0, 247.0)	183.5 (125.0, 260.0)	0.082
Cardiac surgery	241 (2.6%)	136 (2.8%)	73 (2.4%)	32 (2.3%)	0.541
Mechanical Ventilation, n (%)	4829 (51.9%)	2452 (49.7%)	1580 (52.8%)	797 (57.6%)	< 0.001
RRT, n (%)	481 (5.2%)	251 (5.1%)	151 (5%)	79 (5.7%)	0.607
Vasopressors use	2777 (29.8%)	1403 (28.4%)	781 (26.1%)	593 (42.9%)	< 0.001
Diuretics use	5762 (61.9%)	3088 (62.6%)	1770 (59.1%)	904 (65.4%)	< 0.001
Furosemide, mg	40.0 (0.0, 220.0)	40.0 (0.0, 220.0)	40.0 (0.0, 220.0)	50.0 (0.0, 220.0)	0.073
SAPSII	37.0 (29.0, 46.0)	37.0 (29.0, 45.0)	36.0 (28.0, 45.0)	41.0 (32.0, 50.0)	< 0.001
OASIS	34.0 (28.0, 41.0)	34.0 (28.0, 41.0)	34.0 (28.0, 40.0)	37.0 (30.0, 43.0)	< 0.001
SOFA	7.0 (4.0, 10.0)	6.0 (4.0, 9.0)	6.0 (4.0, 9.0)	8.0 (5.0, 12.0)	< 0.001
AKI stage, n (%)					< 0.001
Stage 1	1426 (15.3%)	790 (16%)	472 (15.8%)	164 (11.9%)	
Stage 2	3427 (36.8%)	1819 (36.9%)	1088 (36.3%)	520 (37.6%)	
Stage 3	2255 (24.2%)	1136 (23%)	650 (21.7%)	469 (33.9%)	
Hospital mortality	964 (10.4%)	420 (8.5%)	303 (10.1%)	241 (17.4%)	< 0.001
30-day mortality	905 (9.7%)	391 (7.9%)	285 (9.5%)	229 (16.6%)	< 0.001
1-year mortality	1329 (14.3%)	628 (12.7%)	400(13.4%)	301(21.8%)	< 0.001

*BMI* Body mass index, *HR* Heart rate, *SBP* Systolic blood pressure, *DBP* Diastolic blood pressure, *MBP* Mean blood pressure, *RR* Respiratory rate, *CKD* Chronic kidney disease, *ARDS* Acute respiratory distress syndrome, *sNa* serum sodium, *AG* Anion gap, *BUN* Blood urea nitrogen, *PT* Prothrombin time, *PTT* Partial thrombin time, *INR* International normalized ratio, *WBC* White blood cell, *SAPSII* Simplified acute physiology score, *OASIS* Oxford acute severity of illness, *SOFA* Sequential organ failure assessment, *AKI* Acute kidney injury

### Sodium trajectory patterns and mortality

**Fig. 3:**
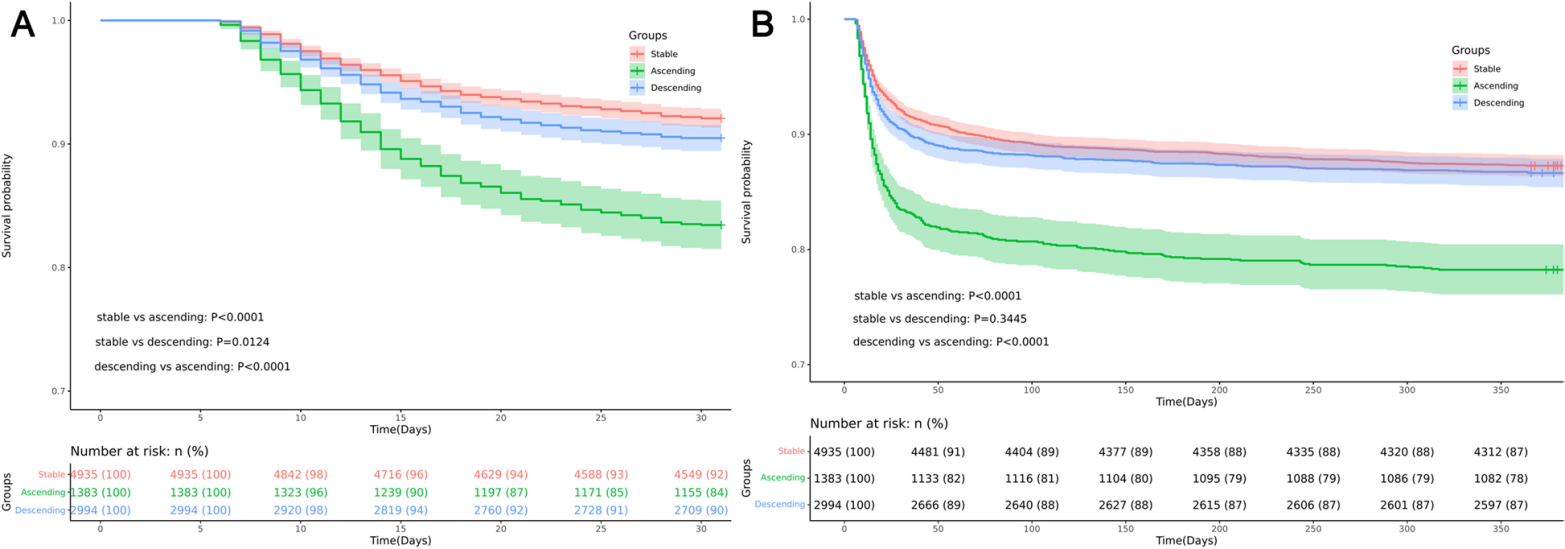
Kaplan–Meier survival estimates of 30-day mortality among each serum sodium trajectory

Among all the enrollees, 905 (9.7%) died within 30 days. The 30-day mortality rates were 7.9% in ST, 9.5% in DS and 16.6% in AS (*p* < 0.001). Besides, the 1-year mortality rates were 12.7%, 13.4% and 21.8% in ST, DS and AS groups, respectively (*p* < 0.001) (Table 1). The Kaplan–Meier curves indicated that the patients with ascending sNa trajectories had the highest risk of 30-day and 1-year mortalities, compared with those in ST and DS groups (shown in Fig. 3). Furthermore, Cox proportional hazard models were employed to determine the association between sNa trajectories and 30-day mortality (Table 2). In crude model, DS (HR = 1.21, 95% CI, 1.04–1.41, *p* = 0.013) and AS (HR = 2.20, 95% CI, 1.87–2.59, *p* = 0.013) were significantly associated with higher 30-day mortality. After stepwise adjustment for demographics, vital signs, comorbidities, mean sNa and diuretics use, DS (HR = 1.22, 95% CI, 1.04–1.43, *p* = 0.015) and AS (HR = 1.68, 95% CI, 1.42–2.01, *p* = 0.013) remained associated with higher mortality (Table 1).

**Table 2:** The association between serum sodium trajectory patterns and 30-day mortality

	Crude model HR (95% CI)	*p*	Model 1 HR (95% CI)	*p*	Model 2 HR (95% CI)	*p*	Model 3 HR (95% CI)	*p*
Stable	reference		reference		reference		reference	
Descending	1.21 (1.04–1.41)	0.013	1.26(1.08–1.46)	0.003	1.22(1.05–1.43)	0.01	1.22 (1.04–1.43)	0.015
Ascending	2.20 (1.87–2.59)	< 0.001	2.14 (1.82–2.52)	< 0.001	1.67 (1.41–1.97)	< 0.001	1.68 (1.42–2.01)	< 0.001

Model 1: adjusted for age, gender, BMI, MBP, heart rate and SpO_2_. Model 2: Model 1 plus CKD, hypertension, diabetes, heart failure, sepsis, stroke, AKI stage and SAPSII, OASIS, SOFA scores. Model 3: Model 2 plus mean sNa and diuretics

*HR* Hazard ratio, *CI* Confidence interval, *BMI* Body mass index, *MBP* Mean blood pressure, *CKD* Chronic kidney disease, *AKI* Acute kidney injury, *SAPSII* Simplified acute physiology score, *OASIS* Oxford acute severity of illness, *SOFA* Sequential organ failure assessment, *sN*a serum sodium

### Subgroup analysis

To further evaluate the robustness of the results of this study, we tested cross-interactions between sodium trajectories and age, gender, hypertension, diabetes, heart failure, CKD, sepsis, vasopressor, diuretics and admission sNa. When patients were stratified by age, gender, diabetes, heart failure and diuretics, the association between the sodium trajectory patterns and 30-day mortality remained similar. However, hypertension (*p* for interaction = 0.032) and CKD (*p* for interaction = 0.040) modified the association between DS trajectory and mortality compared with ST trajectory. Additionally, CKD (*p* for interaction = 0.002), sepsis (*p* for interaction = 0.043) and vasopressor (*p* for interaction = 0.014) modified the association between DS trajectory and mortality compared with ST trajectory (Table 3).

**Table 3:** Subgroup analysis of the associations between sodium trajectories and 30-day mortality

subgroups	No. of patients	Stable Ref	Ascending HR (95% CI)	*p*	***p* for interaction**	Descending HR (95% CI)	*p*	***p* for interaction**
**Age**					0.388			0.474
> 65y	5044	1	2.44 (1.88–3.16)	< 0.001		1.14 (0.88–1.47)	0.334	
≤ 65y	4268	1	2.10 (1.70–2.59)	< 0.001		1.28 (1.05–1.54)	0.012	
**Gender**					0.424			0.741
Male	5250	1	2.33 (1.88–2.90)	< 0.001		1.18 (0.96–1.46)	0.113	
Female	4062	1	2.04 (1.60–2.61)	< 0.001		1.25 (1.00–1.56)	0.051	
**Hypertension**					0.162			0.032
Yes	6030	1	2.02 (1.65–2.48)	< 0.001		1.07 (0.89–1.30)	0.465	
No	3282	1	2.59 (1.96–3.41)	< 0.001		1.52(1.18–1.97)	0.001	
**Diabetes**					0.864			0.286
Yes	2960	1	2.25 (1.69–3.01)	< 0.001		1.07 (0.80–1.42)	0.665	
No	6352	1	2.18 (1.79–2.66)	< 0.001		1.28 (1.07–1.54)	0.007	
**Heart failure**					0.228			0.851
Yes	2914	1	1.91 (1.44–2.55)	< 0.001		1.20 (0.93–1.54)	0.169	
No	6398	1	2.37 (1.94–2.90)	< 0.001		123(1.02–1.49)	0.032	
**CKD**					0.002			0.040
Yes	2023	1	1.40 (1.00–1.97)	0.051		0.93 (0.69–1.26)	0.647	
No	7289	1	2.56 (2.12–3.09)	< 0.001		1.34 (1.12–1.60)	0.001	
**Sepsis**					0.043			0.577
Yes	6333	1	1.88 (1.57–2.24)	< 0.001		1.20 (1.01–1.42)	0.035	
No	2979	1	3.05 (1.98–4.68)	< 0.001		1.35 (0.93–1.94)	0.113	
**Vasopressors**					0.014			0.054
Yes	2777	1	1.59 (1.28–1.98)	< 0.001		1.45 (1.19–1.78)	< 0.001	
No	6535	1	2.41 (1.88–3.10)	< 0.001		1.08 (0.85–1.35)	0.534	
**Diuretics**					0.902			0.078
Yes	5762	1	2.17 (1.78–2.64)	< 0.001		1.35 (1.12–1.62)	0.001	
No	3550	1	2.22(1.66–2.98)	< 0.001		1.00(0.76–1.32)	0.995	

*CKD* Chronic kidney disease

## Discussion

In this retrospective observational study, we identified three sNa trajectory patterns in patients with severe AKI utilitizing GBTM: AS, DS and ST groups. Patients whose serum sodium trajectories were described as ascending or descending were significantly associated with a higher risk of 30-day and 1-year mortalities compared to those with a stable sodium trajectory. This association persisted after adjusting for admission sNa and other potential confounding factors.

Dysnatremia, referring to hyponatremia or hypernatremia, is one of the most common electrolyte disorders in ICU. Numerous studies have shown that dysnatremia is independently associated with poor prognosis, even small changes in sodium concentration could significantly worsen the prognosis [7–10, 16, 17]. For instance, previous studies reported that both borderline dysnatremia on admission and sNa variations of ≥ 6 mEq/L are significantly associated with higher risks of mortality [7, 9, 16, 17]. Collectively, compared to the absolute sNa value at a specific time point, longitudinal sodium fluctuation provided more concise information on disease progression due to its close association to physiological responses, renal dysfunction, and clinical treatment. Yuya et al. conducted a secondary analysis of the AQUAMARINE study and found that sodium dipping, defined as sNa level declined below the baseline level within 48 h, was associated with higher mortality of patients with acute heart failure after adjusting baseline sNa [18]. In contrast to the crude definitions of Yuya, Chewcharat used the GBTM algorithm to identify five distinct sNa trajectories based on longitudinal sNa levels. Compared with stable normonatremia, other sNa trajectory patterns were strongly associated with poor prognosis [13]. Consistent with prior studies, our results revealed that the trajectories of sNa correlated with the prognosis of patients with AKI in ICU, independent of baseline sodium levels, providing new insights into the connection between clinically common dysnatremia and patient prognosis.

Kidney is the main organ involved in water-electrolyte homeostasis and clinically, AKI often coexists with sodium disorders. Although it was documented that coefficient of sodium variation linearly associated with an increased risk of AKI [12], the causal relationship between AKI and dysnatremia remained unclear. In the cases of AKI, the prevalence of dysnatremia ranged from 22.5% to 24.6% and patients with dysnatremia had a higher risk of mortality [12, 19, 20]. Besides, Jonathan reported that it was the patients with trajectory described as uncorrected hypernatremia rather than fluctuating sodium who had the highest risk of mortality by retrospectively analyzing the sodium trajectories of 288 patients [21]. Probably due to sample sizes, its conclusion was not consistent with previous study [13]. There is a contradiction in whether correcting dysnatremia is beneficial to the prognosis. Restoration of initial dysnatremia appeared to benefit in-hospital survival for elderly patients [22]. On the contrary, no additional clinical benefit from correction of dysnatremia for patients undergoing continuous renal replacement therapy (CRRT) as indicated by an observational study [23]. One study even suggested that rapid correction of sodium could even be harmful [24]. In the present study, the GBTM is applied to determine the sNa trajectory patterns of AKI patients based on the patient's sNa values for 7 consecutive days after admission to the ICU. Furthermore, Kaplan–Meier curves and Cox regression models revealed that fluctuation of sNa was associated with the risk of mortality. Combined with our results, sodium fluctuation appeared to be a well-performed indicator of prognosis rather than a therapeutic target.

The mechanism by which sNa trajectory leads to poor prognosis in AKI patients has not been explored. Based on previous studies, we propose the following hypotheses: A previous article on sNa trajectory and poor prognosis suggested that changing aNa creates constant osmotic pressure, the interaction between protein phosphatase 6 (PP6) and apoptosis signal-regulating kinase 3 (ASK3) was affected. and the cell volume recovery system [13], which in turn aggravates AKI by cell dysfunction. On this basis, the disorder of renal function weakens the effective regulation of human electrolytes, leading to further disorder of sNa trajectory, and a vicious circle is formed between the two. In addition, the systemic changes of AKI patients also have an impact on sNa trajectory, especially the changes of the circulatory system, abnormal release of hormone levels, and excessive activation of sympathetic nerves, which may aggravate water and sodium retention. These hypotheses would explain that Ascending sNa trajectory predicts poor prognosis, consistent with our experimental results. Further studies are required to confirm these hypotheses.

Since at least three published studies have used GBTM to identify serum trajectory patterns [13, 21, 24], it is necessary to note the differences between previous studies and ours. On the one hand, the populations enrolled varied: our study included patients diagnosed with AKI within 48 h of ICU admission and previous studies focused on hospitalized patients with AKI, hospitalized patients and patients with heart failure, respectively. On the other hand, Xia determined sodium trajectories based on changes in sodium levels within 48 h of admission, while Chavez and Chewcharat based on multiple in-hospital sodium levels without missing values considered. However, considering the effect of missing values and the possibility of excessive fluctuations in sNa in a short time, we utilized seven consecutive days of sNa to analyze sodium trajectory patterns.

Several limitations must be mentioned in the study. First, our inclusion of patients with AKI occurring within 48 h of ICU admission would cause two problems. On the one hand, some patients would not have baseline serum creatinine data from 7 days earlier. On the other hand, patients with AKI occurring after 48 h would be missed. Second, we used consecutive 1-week longitudinal sNa measurements to determine the sodium trajectories, which improved the reliability of the model. However, the sample sizes were reduced due to the exclusion of patients who did not have their sNa measured for 7 consecutive days, which may underestimate the impact of sNa trajectories on mortality in AKI patients. Third, serum sodium was not adjusted for serum glucose levels and sodium intake was not assessed in this study. Last but not least, due to the observational nature of the investigation, the causal relationship between dysnatremia and clinical outcomes could not be established.

## Conclusion

Using GBTM, AKI patients with dysnatremia could be classified into three distinct serum sodium trajectory patterns with different clinical outcomes, which may be used for risk stratification and prognosis evaluation. Our result revealed the fluctuation of serum sodium in AKI patients, and larger cohort studies and randomized trials are needed to verify our findings.

## Data Availability

Publicly available datasets were analyzed in this study. This data can be found here: https://mimic.mit.edu/.

## References

[1] Clec'h C, Darmon M, Lautrette A, Chemouni F, Azoulay E, Schwebel C (2012). Efficacy of renal replacement therapy in critically ill patients: a propensity analysis. Crit Care.

[2] Lewington AJ, Cerda J, Mehta RL (2013). Raising awareness of acute kidney injury: a global perspective of a silent killer. Kidney Int.

[3] Coca SG, Yusuf B, Shlipak MG, Garg AX, Parikh CR (2009). Long-term risk of mortality and other adverse outcomes after acute kidney injury: a systematic review and meta-analysis. Am J Kidney Dis.

[4] Lee SA, Cozzi M, Bush EL, Rabb H (2018). Distant Organ Dysfunction in Acute Kidney Injury: A Review. Am J Kidney Dis.

[5] Aronson D, Darawsha W, Promyslovsky M, Kaplan M, Abassi Z, Makhoul BF (2014). Hyponatraemia predicts the acute (type 1) cardio-renal syndrome. Eur J Heart Fail.

[6] Hu J, Wang Y, Geng X, Chen R, Zhang P, Lin J (2017). Dysnatremia is an Independent Indicator of Mortality in Hospitalized Patients. Med Sci Monit.

[7] Girardeau Y, Jannot AS, Chatellier G, Saint-Jean O (2017). Association between borderline dysnatremia and mortality insight into a new data mining approach. BMC Med Inform Decis Mak.

[8] Funk GC, Lindner G, Druml W, Metnitz B, Schwarz C, Bauer P (2010). Incidence and prognosis of dysnatremias present on ICU admission. Intensive Care Med.

[9] Sakr Y, Rother S, Ferreira AM, Ewald C, Dunisch P, Riedemmann N (2013). Fluctuations in serum sodium level are associated with an increased risk of death in surgical ICU patients. Crit Care Med.

[10] Marshall DC, Salciccioli JD, Goodson RJ, Pimentel MA, Sun KY, Celi LA (2017). The association between sodium fluctuations and mortality in surgical patients requiring intensive care. J Crit Care.

[11] Lombardi G, Ferraro PM, Calvaruso L, Naticchia A, D'Alonzo S, Gambaro G (2019). Sodium Fluctuations and Mortality in a General Hospitalized Population. Kidney Blood Press Res.

[12] Lombardi G, Ferraro PM, Naticchia A, Gambaro G (2021). Serum sodium variability and acute kidney injury: a retrospective observational cohort study on a hospitalized population. Intern Emerg Med.

[13] Chewcharat A, Thongprayoon C, Cheungpasitporn W, Mao MA, Thirunavukkarasu S, Kashani KB (2020). Trajectories of Serum Sodium on In-Hospital and 1-Year Survival among Hospitalized Patients. Clin J Am Soc Nephrol.

[14] Kellum JA, Lameire N, Group KAGW (2013). Diagnosis, evaluation, and management of acute kidney injury: a KDIGO summary (Part 1). Crit Care..

[15] Zhao GJ, Xu C, Ying JC, Lu WB, Hong GL, Li MF (2020). Association between furosemide administration and outcomes in critically ill patients with acute kidney injury. Crit Care.

[16] Darmon M, Diconne E, Souweine B, Ruckly S, Adrie C, Azoulay E (2013). Prognostic consequences of borderline dysnatremia: pay attention to minimal serum sodium change. Crit Care.

[17] Thongprayoon C, Cheungpasitporn W, Yap JQ, Qian Q (2020). Increased mortality risk associated with serum sodium variations and borderline hypo- and hypernatremia in hospitalized adults. Nephrol Dial Transplant.

[18] Matsue Y, Yoshioka K, Suzuki M, Torii S, Yamaguchi S, Fukamizu S (2017). Prognostic importance of sodium level trajectory in acute heart failure. Heart Vessels.

[19] Krishnamurthy S, Mondal N, Narayanan P, Biswal N, Srinivasan S, Soundravally R (2013). Incidence and etiology of acute kidney injury in southern India. Indian J Pediatr.

[20] Woitok BK, Funk GC, Walter P, Schwarz C, Ravioli S, Lindner G (2020). Dysnatremias in emergency patients with acute kidney injury: A cross-sectional analysis. Am J Emerg Med.

[21] Chavez-Iniguez JS, Maggiani-Aguilera P, Rondon-Berrios H, Kashani KB, Perez-Flores C, Michel-Gonzalez J (2022). Serum sodium trajectory during AKI and mortality risk. J Nephrol.

[22] Chou YH, Lu FP, Chen JH, Wen CJ, Lin KP, Chou YC (2021). Restoration of dysnatremia and acute kidney injury benefits outcomes of acute geriatric inpatients. Sci Rep.

[23] Han SS, Bae E, Kim DK, Kim YS, Han JS, Joo KW (2016). Dysnatremia, its correction, and mortality in patients undergoing continuous renal replacement therapy: a prospective observational study. BMC Nephrol.

[24] Xia YM, Wang S, Wu WD, Liang JF (2023). Association between serum sodium level trajectories and survival in patients with heart failure. ESC Heart Fail.

